# Clinical perspectives in integrating whole-genome sequencing into the investigation of healthcare and public health outbreaks – hype or help?

**DOI:** 10.1016/j.jhin.2020.11.001

**Published:** 2021-03

**Authors:** B.J. Parcell, S.H. Gillespie, K.A. Pettigrew, M.T.G. Holden

**Affiliations:** a Ninewells Hospital and Medical School, Dundee, UK; b School of Medicine, University of St Andrews, St Andrews, UK

**Keywords:** Whole-genome sequencing, Healthcare-associated infections, Typing, Pulsed-field gel electrophoresis, Variable number of tandem repeats, Multi-locus sequence typing

## Abstract

Outbreaks pose a significant risk to patient safety as well as being costly and time consuming to investigate. The implementation of targeted infection prevention and control measures relies on infection prevention and control teams having access to rapid results that detect resistance accurately, and typing results that give clinically useful information on the relatedness of isolates. At present, determining whether transmission has occurred can be a major challenge. Conventional typing results do not always have sufficient granularity or robustness to define strains unequivocally, and sufficient epidemiological data are not always available to establish links between patients and the environment. Whole-genome sequencing (WGS) has emerged as the ultimate genotyping tool, but has not yet fully crossed the divide between research method and routine clinical diagnostic microbiological technique. A clinical WGS service was officially established in 2014 as part of the Scottish Healthcare Associated Infection Prevention Institute to confirm or refute outbreaks in hospital settings from across Scotland. This article describes the authors' experiences with the aim of providing new insights into practical application of the use of WGS to investigate healthcare and public health outbreaks. Solutions to overcome barriers to implementation of this technology in a clinical environment are proposed.

## Introduction

Whole-genome sequencing (WGS) has several advantages over conventional microbiological typing techniques. It can be applied to all micro-organisms (bacteria, fungi, viruses and parasites) and used to analyse their entire genomes [1]. Since Sanger *et al.* sequenced the first complete DNA genome (bacteriophage ϕX174) using the ‘plus and minus’ method in 1977, advances in this field have resulted in increased capacity, reduced costs, and improved speed and reproducibility of results, all of which present an opportunity for WGS to be further incorporated into routine microbiological workflows [[2], [3], [4], [5]]. WGS can be used to pinpoint and track bacteria to a greater degree than traditional typing methods, and has been applied to the investigation of a wide variety of outbreaks [[6], [7], [8]]. WGS has also been used to investigate the emergence and spread of viruses. Due to its portability, nanopore DNA sequencing technology in the form of the MinION (Oxford Nanopore Technologies, Oxford, UK) was utilized for real-time genome sequencing of the Ebola virus disease epidemic in West Africa and yellow fever virus in Brazil [9,10]. The Zika genome has also been sequenced directly from clinical samples using a protocol involving multiplex polymerase chain reaction (PCR) for MinION and Illumina sequencing [11]. More recently, a combined phylogenetic and epidemiological approach was undertaken using Oxford Nanopore and Illumina MiSeq technology to investigate the first 4 weeks of emergence of severe acute respiratory syndrome coronavirus-2 in Scotland [12].

**Figure 1:**
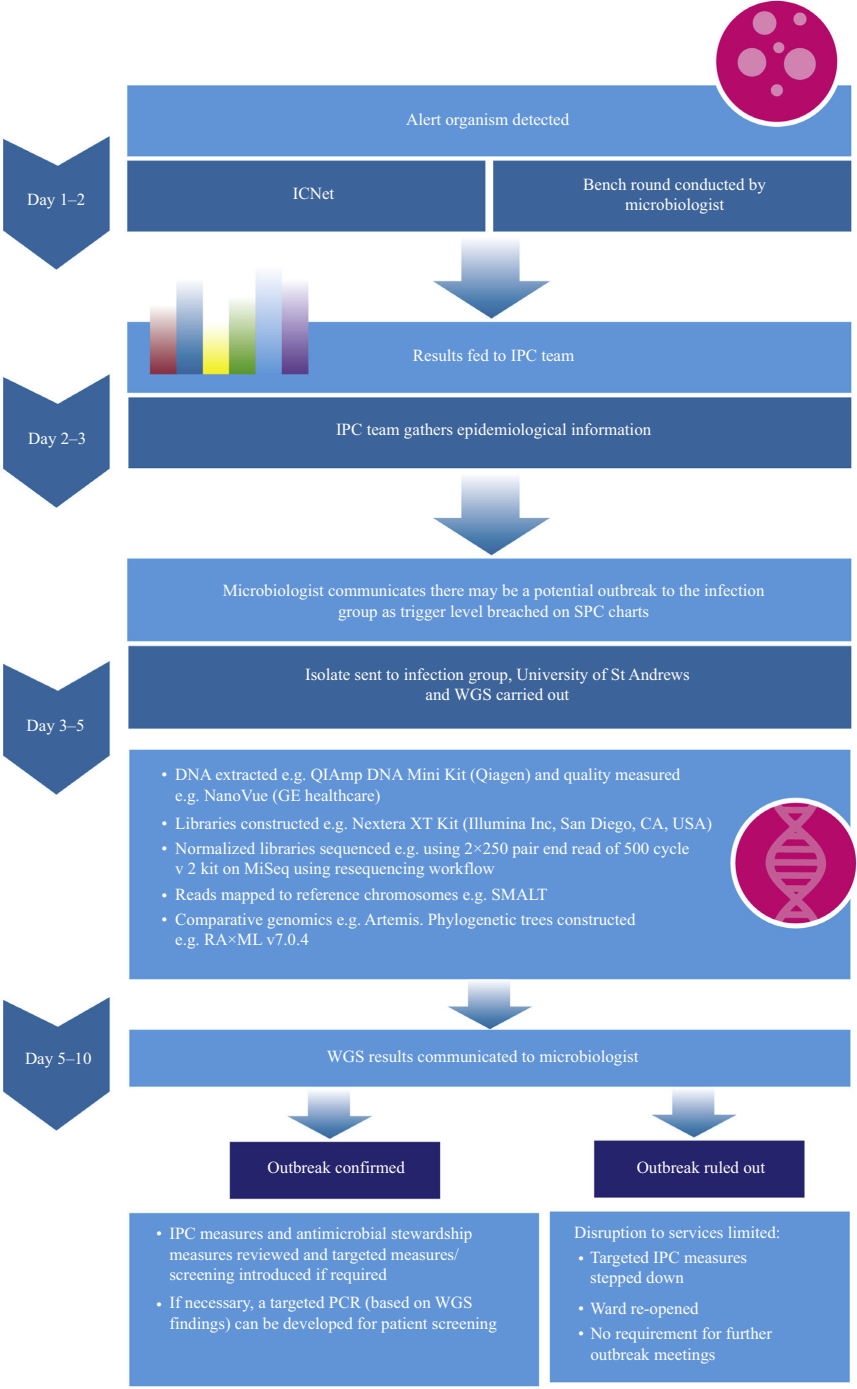
Incorporation of whole-genome sequencing (WGS) into conventional outbreak analysis workflows for in-silico outbreak investigation. IPC, infection prevention and control; SPC, statistical process control; PCR, polymerase chain reaction.

In recent years, reference laboratories have adopted WGS as a standard typing technique for *Escherichia coli*, *Shigella* spp., *Listeria* spp., *Campylobacter* spp., *Staphylococcus aureus, Salmonella* spp. and *Mycobacterium* spp. [13]. In the case of *Salmonella* spp., WGS has emerged as an alternative to the previous gold standard of traditional serology and the Kauffmann–White scheme [14]. Single nucleotide polymorphism (SNP)-based genetic cluster analysis can also be performed to inform epidemiological investigations, such as the UK-wide *Salmonella enteritidis* 25-SNP cluster t25.12 outbreak and national surveillance [15]. Newer bioinformatic methods offer results with improved accuracy, reproducibility and greater resolution. With advances in sequencing technology, there is greater potential to utilize sequencing for real-time outbreak investigations to inform infection prevention and control (IPC) interventions. Greater knowledge and understanding of the transmission of micro-organisms can be generated to inform the best ways to prevent the dissemination of antimicrobial resistance. Consideration must be given as to how best to refine this potentially disruptive technology, optimizing its use in clinical settings so that results are timely, clinically relevant and interpretable by the outbreak management team. This article considers how WGS could be used in the investigation of outbreaks in the future. By using examples from the authors' experience of applying WGS as a targeted IPC tool, this article will illustrate the benefits of harnessing the discriminatory power of WGS in outbreak investigations. In doing so, the aim is to provide pointers towards potential solutions when faced with the challenges brought by this new technology. Over a 5-year period, the authors established a WGS service, with no prior infrastructure, to confirm or refute nosocomial outbreaks in real-time in NHS clinical environments. Clinical specimens were first collected as part of routine care, and initially processed in National Health Service microbiology laboratories using standard methods prior to sequencing by the Infection and Global Health Division, University of St Andrews (Figure 1). Over 3 years, 761 isolates from more than 20 nosocomial outbreaks from health boards across Scotland were sequenced. The pathogens sequenced included: *Acinetobacter baumanii*, *Enterobacter cloacae*, *Enterococcus faecalis*, *Enterococcus faecium*, *E. coli*, *Klebsiella oxytoca*, *Klebsiella pneumoniae*, *Listeria monocytogenes*, *Morganella morganii*, *Mycobacterium abscessus*, *Pseudomonas aeruginosa*, *Enterobacter asburiae, Serratia marcescens*, *S. aureus*, *Staphylococcus epidermidis* and *Streptococcus pyogenes*.

## The value of results with greater granularity due to WGS

It is recognized that routine typing results can obstruct outbreak investigations when results are not rapid, and also when they are unable to show unequivocally whether or not isolates are linked. Pulsed-field gel electrophoresis (PFGE) was previously considered by many to be the gold standard for microbiological typing; however, although it was standardized internationally, it could not decipher evolutionary relationships, or determine whether isolates were truly related in terms of transmission having taken place following identification of a prevalent profile [16,17] Multi-locus sequence typing, which interrogates regions of 400–500 bp from multiple (usually seven) housekeeping genes, is highly reproducible, standardized and easily comparable between laboratories, but lacks discriminatory power for deep epidemiological surveillance [16].

Previously, the authors illustrated the relative discriminatory power of WGS when used for the investigation of an outbreak of *P. aeruginosa* involving nine patients in an adult intensive care unit (ICU) [18]. Both variable number of tandem repeats (VNTR) and PFGE were required to fully determine that transmission had occurred between handwash basin water and two patients. The use of WGS would have provided the necessary information in one step, negating the need for further typing.

## WGS can assist IPCTs when routine typing fails to determine whether or not bacterial isolates are linked due to the commonality of distribution of pathogen types

WGS was used to assist the outbreak team [infection prevention and control team (IPCT) and public and environmental health team] in a hospital outbreak investigation of two cases of invasive listeria infection. *L. monocytogenes* is a notifiable foodborne pathogen which can cause gastroenteritis. Early identification of the source is essential in order to prevent further transmission to patients, staff and the public as listeriosis has a crude mortality rate of 20% and is associated with sepsis, meningoencephalitis and, in pregnant patients, abortion or premature delivery [17]. In this investigation, both patients were immunocompromised and had been admitted to hospital at various points. Routine typing identified the listeria isolates as serotype 4 [clonal complex (CC) 1 and sequence type (ST) 1]. Hospital-wide and ward catering facilities had been inspected and no concerns regarding the practice of food hygiene had been noted. The outbreak team were unsure whether there had been transmission as this was one of the more common serotypes found in clinical isolates (one in six invasive listeria isolates are CC1), and consensus opinion amongst the outbreak management team had been that further catering facility inspections were not required. Both listeria isolates underwent WGS. Reads were initially mapped to the reference chromosome of strain F2365, and SNPs were identified against this. The two isolates were found to be indistinguishable, and therefore highly likely to be epidemiologically linked. In this example, WGS revealed additional information that prompted further action to look again for a common link. The outbreak team repeated hospital kitchen inspections, and as a result of this identified that the handling of salads and meat did not meet national recommendations. Subsequently, hospital catering facilities were closed temporarily until remedial action was undertaken. A further case of invasive serotype 4 listeria infection was identified in a patient 5 months later. WGS ruled this isolate out of the outbreak as it was found to differ by approximately 10,000 SNPs, illustrating that it was too genetically divergent to share a recent common ancestry with the earlier cluster.

## Enhancing the detection of ‘alert organisms’ using genomic analysis

Some countries have an agreed minimum list of micro-organisms that are deemed to pose a risk to patients due to antimicrobial resistance and/or virulence. Flagging up alert organisms such as these allows IPCTs to perform further investigations [19]. The detection of alert bacteria and fungi from patient and/or environmental samples in clinical microbiology laboratories traditionally relies upon the use of phenotypic methods. If the same alert organism is identified from the samples of multiple patients taken within the same timeframe and in a similar place, an outbreak investigation is usually initiated and bacterial isolates will be sent for typing. Statistical process control (SPC) charts can also be used to demonstrate how outbreak strains can accumulate. They display data chronologically and can reveal natural or unnatural variation [20]. However, using approaches which focus solely on alert micro-organisms to detect transmission does not account for the fact that there can be dissemination of genetic elements, such as transposons and plasmids carrying antibiotic resistance genes. This can be a dynamic process, and traditional typing may lack the discriminatory power to identify the genetic lineage of isolates beyond species. This could potentially result in the failure of IPC measures if an alert organism is misidentified or not detected. To explain this point, the benefits of using genomic analysis to detect alert organisms rather than solely accepting results from phenotypic tests was highlighted in the investigation of a seemingly small separate series of vancomycin-resistant *E. faecium* (VREfm) outbreaks. Initially, VREfm was identified in urine cultures from two patients admitted to the same ward of an orthopaedic rehabilitation hospital. Antibiograms were identical, and further screening samples were taken to investigate faecal carriage. Notably, one patient was colonized with two different strains of VREfm – one identified from rectal swabs and another from urine – and each strain was related to an entirely different outbreak cluster in the main hospital. This demonstrated the importance of repeated and sequential patient sampling from different body sites, including multiple colonies for sequencing during an enterococcus outbreak investigation. Over a 2-year period, further positive patients were identified on the surgical high dependency unit and renal ward of the main hospital, totalling 11 cases. Routine PFGE typing of all the VREfm isolates from all patients revealed five separate clusters in total (three ST80 clusters, one ST64 culture and one ST203 cluster). When WGS was applied, it revealed that, in fact, there was only one ST80 cluster, rather than numerous discrete clusters as reported by PFGE. There was a difference of 65–77 SNPs between isolates taken from various patients, suggesting a recent common ancestor (i.e. within the last 5 years). The value of WGS to unravel the transmission pathways was demonstrated when two vancomycin-susceptible *E. faecium* (VSEfm) isolates from two separate patients, identified during a separate VSEfm outbreak in the ICU the preceding year, were linked to the ST64 cluster. This was an unexpected finding, uncovering a hidden transmission event of which the IPCT was not aware. The four isolates were differentiated by only 21 SNPs, suggesting a relatively recent common source. The investigation also revealed that interhospital transmission had occurred between local hospitals and a regional hospital performing renal transplants. These results were produced as part of a real-time VREfm outbreak investigation, and support findings from a retrospective study in the UK in which Raven *et al.* applied WGS to the genetic characterization of 293 bacteraemia isolates [21]. They found that the majority of bacteraemias were hospital-acquired or healthcare-associated, and over 50% of isolates were highly related. In 32% of cases, complex transmissions had occurred over a number of years and across various wards, there was a mixture of vancomycin-resistant and vancomycin-susceptible antibiotic profiles, and this was due to isolates having lost or gained transposons carrying the gene encoding vancomycin resistance (*vanA*) [21]. Taken together, these findings indicate that resistance in enterococci is not stable, and the use of enterococci resistance as a marker for transmission is not reliable. IPCTs therefore need to consider alert organisms in the context of background dissemination of genetic elements in their hospital. For instance, if an increase in the number of cases of enterococcal bacteraemia has been detected, it may be necessary to move quickly to deploy pathogen sequencing to type isolates, irrespective of whether isolates are VSEfm or VREfm.

### Streamlining outbreak investigations in the laboratory

WGS can aid the in-silico investigation of outbreaks by improving turnaround times of results, thereby rapidly streamlining outbreak investigations. It can also be used to replace unnecessary routine laboratory testing, and reduce the transport of isolates to various reference laboratories for traditional typing. Carbapenemase-producing Enterobacterales are notoriously challenging to detect in clinical laboratories, and multiple methods are routinely used to detect them. In an outbreak involving three renal patients infected with *bla*_KPC_*-*positive ST258 *K. pneumoniae*, WGS was found to negate the need for screening by various multiplex PCR assays in two different laboratories and VNTR analysis. Additionally, WGS revealed that one of the samples was mixed with an ST3 *E. coli* which had not been identified on routine testing.

## Ruling out outbreaks with WGS

WGS can also be used by IPCTs to rule out outbreaks, avoiding disruption to services by removing the need for staff and time-consuming outbreak meetings. If WGS can be used to rule out an outbreak, staff can focus on preventive measures and tasks which could stop the occurrence of outbreaks. There would be additional benefits to the hospital and patients as outbreak control measures could be stopped, wards could open, and patients would not require screening. Extra cleaning regimes, such as twice-daily cleaning of commonly touched surfaces with chlorine disinfectant, with increased domestic staff input would not be necessary. In one example illustrating these points, a doctor highlighted that they had noticed three separate patients who had extended-spectrum beta-lactamase-positive *E. coli* urine cultures with identical antibiograms. The patients resided in a residential care home, and the doctor was concerned that this may represent a breakdown in IPC measures. An outbreak was suspected by the IPCT as an alert organism had been detected from the samples of three separate patients taken within the same timeframe. In this situation, the three isolates underwent WGS and were mapped to ST43 (ST131) reference chromosome sequence from an isolate that originated in the UK. Looking at the core genome, differences ranged from 99 SNPs to 162 SNPs, providing reassurance that this was not an outbreak.

## Using WGS to uncover new resistance mechanisms

An essential part of IPC is horizon scanning: identifying new threats including new pathogens and resistance mechanisms. Aside from its utility for high-resolution SNP-based typing, WGS can capture the whole-genome inventory of an organism, and can therefore be used as a vital tool for the investigation of emerging resistance mechanisms. *optrA* is an ABC transporter gene, first reported in 2015, that encodes resistance to oxazolidinones such as linezolid via active efflux [22]. National resistance alerts have been issued highlighting the risk to public health as this new resistance mechanism is plasmid-mediated and could potentially transfer to other strains, species and genera present on the skin and gut of humans and animals [23]. In 2016, an IPCT investigation was undertaken as Public Health England's Antimicrobial Resistance and Healthcare Associated Infections Reference Unit confirmed a patient had a urine sample which was positive for *optrA* linezolid-resistant enterococcus. A retrospective search of the hospital's historical laboratory culture results identified two further patients with *E. faecalis* isolates that were linezolid resistant, identified in 2014 and 2015. WGS was applied to investigate these isolates and found that both were positive for the *optrA* gene. A review of the epidemiology revealed that all patients had urinary tract infections. WGS was valuable in this situation as it identified that the isolates were of three distinct sequence types (ST480, ST19 and ST330), confirming that resistance had emerged separately in the *E. faecalis* population.

## Barriers and facilitators to translating the promise of WGS into clinical practice

This article has identified some of the barriers and facilitators to translating the promise of WGS into clinical practice in a system-based manner. The first barrier to integration of WGS into conventional outbreak analysis in any clinical microbiology laboratory is infrastructure. Options include placing equipment at national reference laboratories, at hub sites (e.g. large teaching hospitals) or outsourcing to private laboratories. However, the continued progress of sequencing technology has enabled clinical microbiology laboratories to come a step closer to performing low-cost WGS themselves, using simple bench-top technology and user-friendly library preparation protocols [24]. Benefits of placing facilities closer to patients include streamlining of work (replacement of multiple tests and reduction in sending samples away for confirmatory testing) which could result in reduced turnaround times. However, if WGS is incorporated into local teaching hospital facilities, there would need to be investment in appropriately trained staff, equipment and data analysis. It should also be considered that the future of clinical microbiology is changing and less conventional microbiology is being undertaken. There has been increased use of molecular methods such as PCR, point-of-care testing and automation. Staff skill mix is changing; for instance, consultant microbiologists are carrying out less authorization of routine results, and more staff are familiar with DNA extraction and PCR techniques. This could present an opportunity for WGS to be utilized within clinical microbiology services. To increase the success of a business case, the potential impact of WGS on patient management should be included. This could include its impact on antimicrobial stewardship, IPC measures, outbreak investigation and subsequent impact on services. To further strengthen a business case, WGS facilities could be shared with other departments, such as human genetics or a university department, provided that appropriate approvals are in place. Regardless of location, laboratories need to assure the quality of WGS and validate their methods [25,26]. It is also essential that laboratories are accredited by the United Kingdom Accreditation Service.

Additional guidance for sharing genomic data needs to be developed so that patient privacy is maintained yet genomic sequences can be shared and used in an early warning system for outbreaks. Thought must be given to producing an actionable result within a useful timeframe, and it is essential for results to be clear and meaningful so they can be interpreted by staff in terms of the clinical picture. Therefore, curriculums for microbiology and infection, public health and IPC training should also incorporate basic training on interpretation of WGS results.

**Figure 2:**
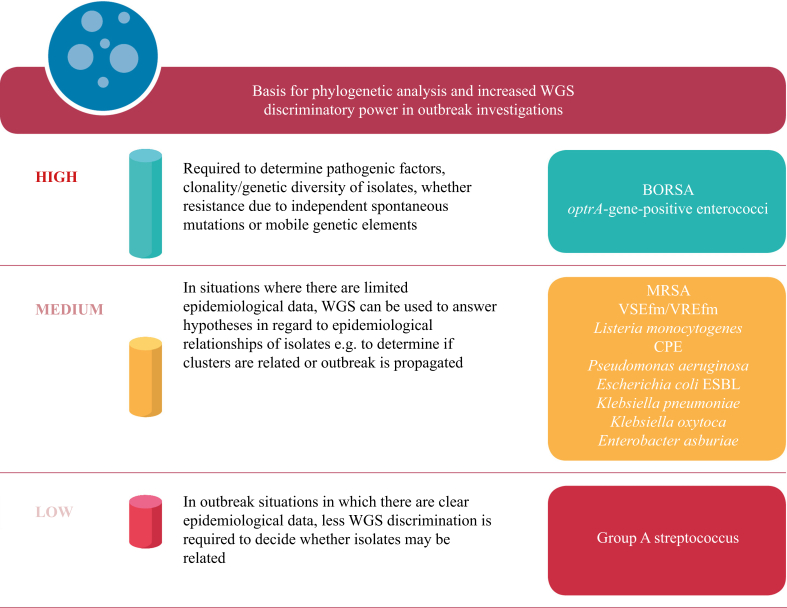
Basis for phylogenetic analysis and increased whole-genome sequencing (WGS) discriminatory power in outbreak investigations. BORSA, borderline oxacillin-resistant *Staphylococcus aureus*; MRSA, meticillin-resistant *S. aureus*; VSEfm, vancomycin-susceptible *Enterococcus faecium*; VREfm, vancomycin-resistant *E. faecium*; CPE, carbapenemase-producing Enterobacterales; ESBL, extended-spectrum beta-lactamase.

Based on the authors' experience of using real-time WGS for outbreak investigations, instances in which increased WGS discriminatory power and phylogenetic analysis is required have been identified (Figure 2). There continues to be a significant cost to sequencing, and using WGS for the investigation of all outbreaks is not likely to be feasible for the foreseeable future. The discriminatory power of WGS was particularly valuable in outbreak situations with new or unusual resistance mechanisms. The majority of outbreaks required a medium level of WGS discriminatory power to determine if isolates were related. In instances where the epidemiology demonstrated that there was likely to be an outbreak, less WGS discrimination was required.

**Figure 3:**
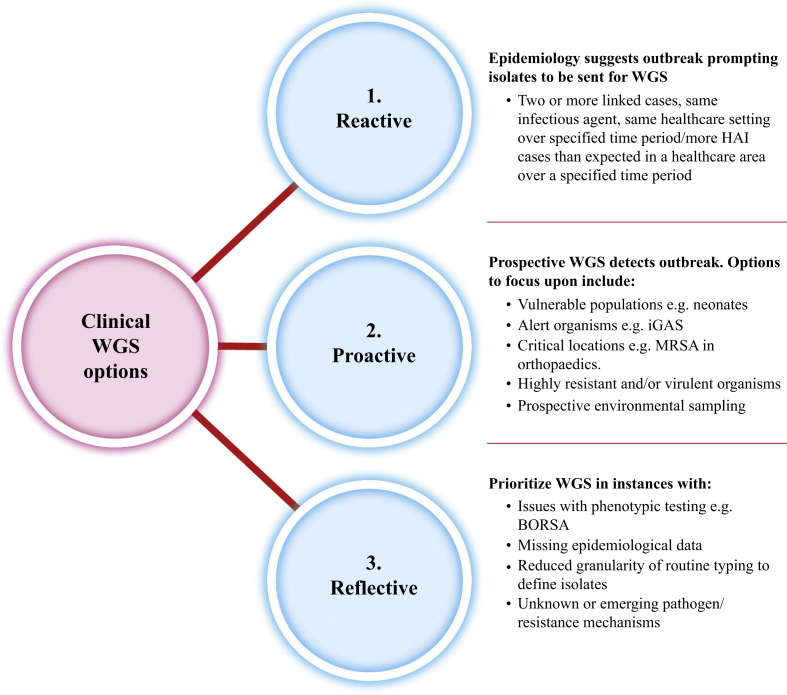
Recommendations for using whole-genome sequencing (WGS) for the detection of nosocomial outbreaks. HAI, hospital-acquired infection; MRSA, meticillin-resistant *Staphylococcus aureus*; BORSA, borderline oxacillin-resistant *S. aureus.*

Considering the findings, it is suggested that there could be different approaches to the use of WGS for outbreak detection (Figure 3). The first approach involves a reactive automated response to epidemiology suggestive of an outbreak. In this instance, phenotypic methods may suggest that there is an outbreak and isolates could then undergo WGS. An alternative to this is a proactive approach in which WGS is used to detect an outbreak regardless of epidemiology. In these instances, users of WGS could prospectively sequence select populations of patients who may be vulnerable to infection who may be from a critical location, such as a neonatal ICU, or focus upon sequencing a highly resistant or virulent organism from patient samples or the environment. Sequencing a specific group of micro-organisms that cause nosocomial infections in a complete geographic area can give a high-resolution view of the pathogen population that can pinpoint the genetic basis of resistance and spread of the pathogen. This would represent a shift in the identification of outbreaks. A reflective approach to outbreak investigations could include the use of WGS in defined instances, when there is missing epidemiological data or in scenarios in which phenotypic testing lacks granularity.

**Figure 4:**
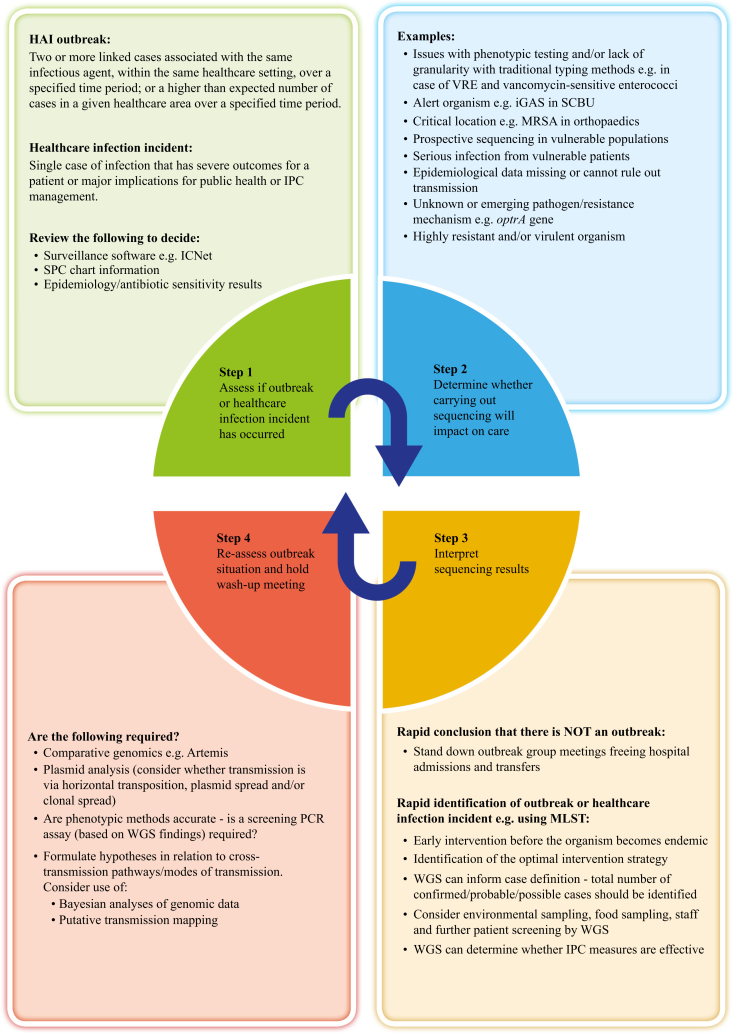
Whole-genome sequencing (WGS) clinical decision aid. HAI, hospital-acquired infection; IPC, infection prevention and control; SPC, statistical process control; MRSA, meticillin-resistant *Staphylococcus aureus*; PCR, polymerase chain reaction; VRE, vancomycin-resistant enterococci; SCBU, special care baby unit; MLST, multi-locus sequence typing; iGAS, invasive group A streptococcal disease.

A clinical decision aid (Figure 4) has been developed to assist clinicians in selecting isolates for sequencing, and actions following the production of results. The speed at which outbreaks emerge differs by organism and in accordance with the micro-organism's ecology or clinical setting. Clustered organisms can arise sufficiently far apart in time or location that they are not identified; therefore, local surveillance systems should be set to have a trigger/threshold that prompts IPC action. It is suggested that the process of outbreak identification should be automated by developing systems that function without human coordination with set ‘action line’ rules. This can be brought about by collecting epidemiological data, using SPC charts and implementing outbreak surveillance software such as ICNet. Care needs to be taken in relation to the interpretation of results, particularly with regard to the meaning of SNP differences. These need to be considered in the context of pathogen genome stability and the environment. For instance, the literature reports that various SNP differences have been found during the investigation of listeria outbreaks, with diversity ranging from zero to five SNPs and, in some outbreaks, up to 42 SNPs [27]. At the time when the authors investigated the listeria outbreak, Public Health England observed that listeria isolates in outbreaks linked to a single food premises can be as many as 20 SNPs apart, which is in contrast to findings in verocytotoxigenic *E. coli* and salmonella incidents, where only isolates within five SNPs of each other would be considered to be linked. This is because *Listeria* spp. can remain as environmental contaminants in premises over many years (G. Hawkins, Health Protection Scotland, Personal Communication, 1 September 2016).

WGS can be an asset at every stage of outbreak management, assisting IPCTs in the formulation of case definitions and supporting or refuting hypotheses in relation to lines of transmission. When coupled with epidemiological data, WGS can provide the ultimate discrimination of results, enabling IPCTs to carry out outbreak investigations efficiently and effectively. Its value also lies in testing the effectiveness of IPC measures and being able to rule out outbreaks, which negates the requirement for outbreak meetings and disruption to healthcare services. With the global threat of dissemination of antimicrobial resistance, WGS is a valuable tool that should be used to generate greater understanding of the development of new resistance mechanisms and dissemination of resistance elements. In the authors' opinion, the benefits of using WGS for outbreak investigation that have been encountered since establishment of a clinical WGS service in 2014 have far outweighed the efforts to confront the challenges of implementing this technology into routine care.
